# Supplementary material for: Long-term outcomes of ivermectin-albendazole versus albendazole alone against soil-transmitted helminths: Results from randomized controlled trials in Lao PDR and Pemba Island, Tanzania
Source: PLoS Negl Trop Dis. 2021 Jun 30;15(6):e0009561. doi: 10.1371/journal.pntd.0009561 (PMC8277064; doi:10.1371/journal.pntd.0009561)
Supplement: S1 Text — (PDF) [file pntd.0009561.s001.pdf]

S1 Text. Household questionnaire.

Date: |__|__|/|__|__|/2018<br>Place: ______________________<br>Interviewer: ______________________	**Household ID:**<br>**|__|__|__|__|**
**Participant ID: |__|__|__|__|**<br>Sex: **|__|** [M/F] Age: **|__|__|**	

A) Socioeconomic factors

A1. Which of the following items do you have in your household/do you own?

Note: only owned by the respective household NOT other family members living in other households.

MULTIPLE CHOICES

Item		Item	
**1. Books/newspapers**	☐ Yes ☐ No	11. Charcoal for cooking	☐ Yes ☐ No
**2. Radio**	☐ Yes ☐ No	12. Gas for cooking	☐ Yes ☐ No
**3. Television**	☐ Yes ☐ No	13. Cell phone	☐ Yes ☐ No
**4. Video/DVD player**	☐ Yes ☐ No	14. Solar panels	☐ Yes ☐ No
**5. Fan**	☐ Yes ☐ No	15. Air conditioning (AC)	☐ Yes ☐ No
**6. Refrigerator/Freezer**	☐ Yes ☐ No	*If in possession of Television:*	
**7. Bike**	☐ Yes ☐ No		
**8. Motorbike**	☐ Yes ☐ No	16. TV signal: Cable	☐ Yes ☐ No
**9. Car**	☐ Yes ☐ No	17. TV signal: Box receiver	☐ Yes ☐ No
**10. Electricity (cable)**	☐ Yes ☐ No	18. TV signal: Satellite dish	☐ Yes ☐ No

A2. What are the walls of your house(s) made of?

Note: If they have several houses give priority to the best equipped building.

MULTIPLE CHOICES

**1. Bamboo/palm leaves**	**☐ Yes ☐ No**	**4. Clay & stones/concrete mix**	**☐ Yes ☐ No**
**2. Wood (planks or boards)**	☐ Yes ☐ No	5. Concrete/Bricks/Geo-concrete	☐ Yes ☐ No
**3. Wooden frame & clay only**	☐ Yes ☐ No	6. Other: __________________	☐ Yes ☐ No

A3. What is the floor of your house(s) made of?

Note: If they have several houses give priority to the best equipped building.

MULTIPLE CHOICES

**1. Soil**	**☐ Yes ☐ No**	**5. Concrete/Geo-concrete**	**☐ Yes ☐ No**

**2. Clay**	☐ Yes ☐ No	6. Tiles/tiles-concrete mix floor	☐ Yes ☐ No
**3. Bamboo/palm leaves**	☐ Yes ☐ No	7. Other: ________________	☐ Yes ☐ No
**4. Wood (planks or boards)**	☐ Yes ☐ No		

A4. What is the roof of your house(s) made of?

Note: If they have several houses give priority to the best equipped building.

MULTIPLE CHOICES

**1. Plants *(bamboo, grass, palm leaves)***	**☐ Yes ☐ No**	**4. Corrugated metal *(aluminum, zinc)***	**☐ Yes ☐ No**
**2. Wood (planks or boards)**	☐ Yes ☐ No	5. Tiles	☐ Yes ☐ No
**3. Plastic**	☐ Yes ☐ No	6. Other: ________________	☐ Yes ☐ No

B) Presence of sanitation and water structures at household level

B1. Do you dispose of any shower at your household? And what material is its floor made of?

Note: Verify that it is their private shower and not a common/shared shower belonging to somebody else. SINGLE CHOICE

☐ 1. No shower
☐ 2. Shower with sand/earth floor
☐ 3. Shower with gravel floor
☐ 4. Shower with tiles/tiles-concrete mix floor
☐ 5. Shower with concrete floor
☐ 6. Other: ___________________
☐ 7. Common/shared shower

B2. Do you dispose of any latrine/toilet at your household?

Note: Verify that it is their private latrine/shower and not a common/shared latrine/toilet belonging to somebody else. SINGLE CHOICE

☐ Yes, a private latrine
☐ Yes, a shared/common latrine
☐ No
