# Supplementary material for: Long-term outcomes of ivermectin-albendazole versus albendazole alone against soil-transmitted helminths: Results from randomized controlled trials in Lao PDR and Pemba Island, Tanzania
Source: PLoS Negl Trop Dis. 2021 Jun 30;15(6):e0009561. doi: 10.1371/journal.pntd.0009561 (PMC8277064; doi:10.1371/journal.pntd.0009561)
Supplement: S1 Table — (PDF) [file pntd.0009561.s002.pdf]

	Time point post-treatment	Albendazole monotherapy	Ivermectin-albendazole	Difference between the two treatments (%)
**Lao PDR**				
Ascaris lumbricoides				
N. of randomized participants (%)		274 (100)	275 (100)	
N. of co-infected participants (%)		112/274 (40.9)	96/275 (34.9)	
N. of participants negative/N. surveyed (%)	14-21 days	193/194 (99.5)	213/213 (100)	0.5
	6 months	172/232 (74.1)	184/234 (78.6)	4.5
	12 months	166/205 (80.1)	170/208 (81.7)	0.8
N. of new infections/N. surveyed (%)	6 months	60/232 (25.9)	50/234 (21.4)	
	12 months	39/205 (19.0)	38/208 (18.3)	
EPG geometric mean	Baseline	24.4	13.4	
	14-21 days	0.0	0.0	
	6 months	5.3	3.7	
	12 months	3.5	3.0	
EPG aritmethic mean	Baseline	5150.9	3221.2	
	14-21 days	1.0	0.0	
	6 months	2934.7	1257.4	
	12 months	2143.6	1430.7	
ERR geometric (95% CI)	14-21 days	99.9	100	0.1
Extended ERR	6 months	82.2	75.8	-6.4
	12 months	87.3	77.0	-10.3
**Hookworm**				
N. of co-infected participants (%)		250/274 (91.2)	253/275 (92.0)	
N. of participants negative/N. surveyed (%)	14-21 days	114/194 (58.8)	132/213 (62.0)	3.2
	6 months	111/232 (47.8)	117/234 (50.0)	2.2
	12 months	126/205 (61.5)	146/208 (70.2)	
N. of new infections/N. surveyed (%)	6 months	121/232 (52.2)	117/234 (50.0)	
	12 months	79/205 (35.5)	62/208 (29.8)	
EPG geometric mean	Baseline	454.0	471.1	
	14-21 days	6.6	5.5	

	6 months	13.7	12.2	
	12 months	5.4	3.6	
EPG aritmethic mean	Baseline	1503.6	1’529.0	
	14-21 days	136.0	118.2	
	6 months	390.4	304.4	
	12 months	446.9	154.7	
ERR geometric (95% CI)	14-21 days	99.0	98.8	-0.2
Extended ERR	6 months	96.6	97.7	1.1
	12 months	98.8	99.2	0.4
S. stercoralis[a]				
N. of co-infected participants (%)		7/273 (2.6)	10/264 (3.6)	
N. of participants negative/N. surveyed (%)	14-21 days	190/193 (98.5)	213/213 (100)	
	6 months	227/232 (97.8)	231/232 (99.6)	
	12 months	202/205 (98.5)	206/208 (99.0)	
N. of new infections/N. surveyed (%)	6 months	5/232 (2.2)	1/231 (0.4)	
	12 months	3/205 (1.5)	2/208 (1.0)	
**Pemba Island**				
Ascaris lumbricoides				
No. of randomized participants		305 (100)	308 (100)	
N. of co-infected participants (%)		74/305 (24.3)	90/308 (29.2)	
N. of participants negative/N. surveyed (%)	14-21 days	291/293 (99.3)	286/288 (99.3)	0
	6 months	205/282 (72.7)	215/276 (77.9)	5.2
	12 months	204/264 (77.3)	185/251 (73.7)	-3.5
N. of new infections/N. surveyed (%)	6 months	77/282 (27.3)	61/276 (22.1)	
	12 months	60/264 (22.7)	66/251 (26.3)	
EPG geometric mean	Baseline	6.4	10.1	
	14-21 days	0.1	0.1	
	6 months	6.6	4.8	
	12 months	4.5	5.4	
EPG aritmethic mean	Baseline	2275.7	2323.8	
	14-21 days	24.2	117.0	
	6 months	1231.1	984.9	

	12 months	999.1	818.1	
ERR geometric (95% CI)	14-21 days	99.1	99.4	0.3
Extended ERR	6 months	-5.7	47.4	53.1
	12 months	25.0	48	23.0
**Hookworm**				
No. of randomized participants		305 (100)	308 (100)	
N. of co-infected participants (%)		53/305 (17.4)	42/308 (13.6)	
N. of participants negative/N. surveyed (%)	14-21 days	281/293 (95.9)	275/288 (95.5)	-0.4
	6 months	262/282 (92.9)	262/276 (94.9)	2.0
	12 months	250/264 (94.7)	231/251 (92.0)	-2.6
N. of new infections/N. surveyed (%)	6 months	20/282 (7.1)	14/276 (5.1)	
	12 months	14/264 (5.3)	20/251 (8.0)	
EPG geometric mean	Baseline	1.2	0.8	
	14-21 days	0.2	0.2	
	6 months	0.3	0.2	
	12 months	0.3	0.4	
EPG aritmethic mean	Baseline	37.3	29.0	
	14-21 days	5.2	14.0	
	6 months	6.7	12	
	12 months	9.0	18.4	
ERR geometric (95% CI)	14-21 days	84.8	71.6	-13.2
Extended ERR	6 months	72.2	73.2	1.0
	12 months	74.5	59.3	-15.2

Note:

New infections were defined for infections with *A. lumbricoides* or hookworm at 6 or 12 months post-treatment, as all participants, per study design, were po

EPG = egg per gram of stool

[a] not all participants were assessed for *S. steroralis* co-infection, the total number examined is indicated as number surveyed
