# Supplementary figures and images for: Long-term outcomes of ivermectin-albendazole versus albendazole alone against soil-transmitted helminths: Results from randomized controlled trials in Lao PDR and Pemba Island, Tanzania
Source: PLoS Negl Trop Dis. 2021 Jun 30;15(6):e0009561. doi: 10.1371/journal.pntd.0009561 (PMC8277064; doi:10.1371/journal.pntd.0009561)

**S1 Fig. Reported open defecation practices.**

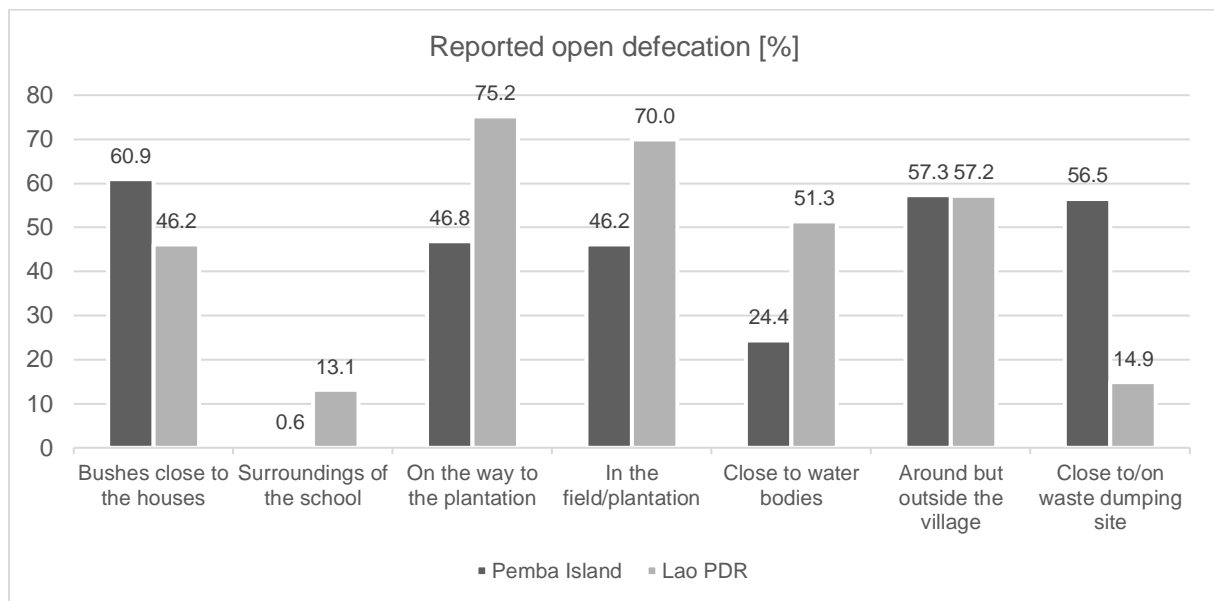

Supplement: S1 Fig — Multiple choice question that was asked to one adult household member per screened household. (PDF) [file pntd.0009561.s003.pdf]
